# Signaling responses to high and moderate load strength exercise in trained muscle

**DOI:** 10.14814/phy2.14100

**Published:** 2019-05-14

**Authors:** Evgeny A. Lysenko, Daniil V. Popov, Tatiana F. Vepkhvadze, Anna P. Sharova, Olga L. Vinogradova

**Affiliations:** ^1^ Laboratory of Exercise Physiology SSC RF Institute of Biomedical Problems RAS Moscow Russia; ^2^ Faculty of Fundamental Medicine M.V. Lomonosov Moscow State University Moscow Russia

**Keywords:** Leg press, mTORC1, muscle biopsy, translation

## Abstract

We examined signaling responses in the skeletal muscle of strength athletes after strength exercises under high and moderate load. Eight trained male powerlifters were recruited. The volunteers performed four sets of leg presses to volitional fatigue using a moderate load (65% 1‐repetition maximum [1RM]) for one leg, and a high load (85% 1RM) for the contralateral leg. The work volume performed by the leg moving a moderate load was higher than that of the contralateral leg moving a high load. Biopsy of the *m*. *vastus lateralis* was performed before, and at 1, 5, and 10 h after, cessation of exercise. Phosphorylation of p70S6k^Thr389^, 4E‐BP1^Thr37/46^, and ACC^S^ ^er79^ increased after moderate load exercises, whereas phosphorylation of ERK1/2^Thr202/Tyr204^ increased, and that of eEF2^Thr56^ decreased, after high load exercises. Exercise under a moderate load and a high work volume activated mTORC1‐dependent signaling in trained skeletal muscle, whereas exercise under a high load but lower work volume activated the MEK‐ERK1/2 signaling cascade and eEF2.

## Introduction

Strength training is practiced widely by those participating in sports, as well as by those undergoing rehabilitation or suffering illness. The main aim is to maintain or increase muscle mass and strength. At first glance, the most significant variable in strength training is exercise load. However, strength training performed to volitional fatigue using high and moderate loads leads to similar levels of skeletal muscle hypertrophy in untrained (Popov et al. 2006; Mitchell et al. 2012) and strength trained men (Schoenfeld et al. 2015; Morton et al. 2016). It was suggested that this effect is associated with comparable muscle fiber recruitment and comparable rates of protein synthesis when performing exercises to fatigue under varying load (Burd et al. 2010).

Mammalian target of rapamycin complex 1 (mTORC1) is a key regulator of protein synthesis in skeletal muscle. Activation of mTORC1 in response to strength exercise results in phosphorylation of the translation initiation regulators ribosome protein S6 kinase (p70S6k) and Eukaryotic translation initiation factor 4E (eIF4E) ‐ binding protein 1 (4E‐BP1), and subsequent acceleration of protein synthesis (Goodman 2014). A specific inhibitor of mTORC1, called rapamycin, blocks increases in protein synthesis in the muscles of young recreationally active men completely during the first 2 h of recovery after strength exercise (Drummond et al. 2009). In addition, in recreationally active men there is a correlation between increased p70S6k^Thr389^ phosphorylation and increased protein synthesis after acute strength exercise (Kumar et al. 2009), as well as between increased p70S6k^Thr389^ phosphorylation and muscle hypertrophy after strength training (Terzis et al. 2008). The degree of mTORC1 activation may depend on the exercise load (Kumar et al. 2009) and work volume (Terzis et al. 2010) in recreationally active men. By contrast, although the increase in the rate of protein synthesis in muscles of those men is similar after strength exercise performed to volitional fatigue with 30% or 90% of 1‐repetition maximum (1RM) loads, activation of the mTORC1‐dependent signaling is different (Burd et al. 2010). It could be suggested that a similar increase in the rate of protein synthesis after strength exercise performed to volitional fatigue under different loads, and the similar increases in hypertrophy caused by strength training performed to volitional fatigue under different loads are mediated by different patterns of activation of anabolic and, possibly, catabolic signaling pathways. However, the characteristics that determine activation of anabolic and catabolic signaling pathways after strength exercise performed to volitional fatigue under different loads are unclear.

Here, we hypothesized that strength exercises performed to volitional fatigue under different loads activate different signaling cascades to varying degrees, and that this activation is sensitive to work volume and/or exercise load. To test this, we examined signal responses in skeletal muscle of strength athletes after strength exercises performed to volitional fatigue under high and moderate loads.

## Methods

### Subjects

Eight trained male competitive power lifters (median age 29 years [interquartile range (IQR) 24–35]; height 176 [172–182] cm; body mass 92 [83–102] kg; BMI 29.2 [27.0–32.8] kg/m^2^, powerlifting training experience 11 [9–16] years, the frequency of training three times a week, the maximum performance when squatting with a barbell without equipment 225 [200–235] kg) were recruited. All volunteers routinely used a high protein diet. They competed under the auspices of the International Powerlifting Federation and were regularly tested for doping. Subjects were informed of the purpose of the study, the experimental procedures, and potential risks, and all provided written consent to participate. The study was approved by the Human Ethics Committee of the Institute and complied with the guidelines of the Declaration of Helsinki.

### Procedures

#### Preliminary experiments

During the first visit to the laboratory, participants performed five sets with a gradually increasing load (familiarization exercises) using seated leg press machine (SSC RF Institute of Biomedical Problems RAS, Russia) with pneumatic drive (FESTO). Before the second visit, all participants refrained from any physical activity for 3 days. During the second visit, the 1RM for each leg was determined. For this purpose, participants performed a warm‐up with a light weight and then performed one repetition with a gradually increasing weight until they were unable to move the weight. The last proper repetition was designated the 1RM; volunteers performed no more than five attempts to prevent fatigue. After a 15 min rest, the exercise session (three sets to exhaustion with a load corresponding to 65% 1RM for one leg and 85% 1RM for the other leg) was performed to ensure familiarization with the load to be used during the experiment. The choice of legs for different exercise loads was randomized in a counter‐balanced manner.

#### Main study

All participants refrained from any physical activity for 3 days before the day of the experiment. Participants arrived at the laboratory at 07:00 and consumed a standardized light breakfast (1135 kJ; 8 g protein, 50 g carbohydrate, 4 g fat). From 09:00, they laid on a couch for half an hour. Next, venous blood was obtained from the *v. intermedia cubiti* via a catheter and microbiopsy samples (Hayot et al. 2005) were taken from the *m*. *vastus lateralis* of each leg under local anesthesia (2 mL 2% lidocaine). After the biopsy, the volunteers performed a warm‐up session with a light weight, followed by four sets of leg press to fatigue with a moderate load (65% 1RM) for one leg and the four sets of leg press to fatigue with a high load (85% 1RM) for the contralateral leg. Sets were performed alternately with rest intervals of 2 min: i.e., each leg received 4 min rest between consecutive sets. Venous blood samples were obtained immediately after cessation of exercise and again 15 min later. Biopsy of the *m*. *vastus lateralis* was performed at 1, 5, and 10 h after cessation of exercise. For each subsequent biopsy, a new puncture was made 2 cm proximal to the previous one. Muscle samples were quickly blotted with gauze to remove superficial blood, frozen in liquid nitrogen, and stored at −80°C until further analysis. A standardized meal (4849 kJ; 37 g protein, 126 g carbohydrate, and 67 g fat) was provided at 75 min and at 5.5 h after exercise.

#### Measuring of blood lactate and hormone levels

Lactate levels in venous blood were measured immediately after collecting using a Biosen C‐line analyzer (EKF Diagnostics, Germany). The blood was collected in tubes with EDTA and centrifuged (1500*g*, 15 min); the supernatant aliquots were frozen at −80°C. Blood cortisol and testosterone levels were evaluated in enzyme‐linked immunosorbent assays (ELISA) using commercial ELISA‐Cortisol and ELISA‐Testosterone Kits (ImmunoTek, Moscow, Russia). Aliquots were used once. All measurements were made in duplicate; the coefficient of variation not exceeding 5%.

#### RNA extraction

Frozen samples (~20 mg) were sectioned (20 *μ*m thick) using an ultratome (Leica Microsystems) and RNA extracted using an RNeasy Mini Kit (Qiagen). RNA concentration and purity were measured using a NanoDrop 2000 spectrophotometer (Thermo Scientific). After DNase treatment (Fermentas), cDNA was synthesized from 1 *μ*g of total RNA using the MMLV Reverse Transcriptase kit (Evrogen).

#### Real‐time polymerase chain reaction

Real‐time polymerase chain reaction (PCR) was performed using the Rotor‐Gene Q cycler (Qiagen). The annealing temperature was optimized for each primer pair. The thermal profile included an initial heat denaturing step at 95°C for 5 min, followed by 45 cycles of denaturation at 95°C for 15 sec, annealing (56–60°C) for 30 sec, and extension at 72°C for 30 sec. Amplified genes were quantified via fluorescence using the EvaGreen Master Mix (Syntol). The specificity of amplification was monitored via melting curve analysis and agarose gel (1%) electrophoresis. Each sample was run in triplicate and a nontemplate control was included in each run. Target gene mRNA expression levels were calculated using the efficiency‐corrected ∆Ct method with the formula: $\sqrt[{2}]{{\left(1+E_{ref1}\right)}^{Ct_{\mathrm{ref}1}}\times {\left(1+E_{\mathrm{ref}2}\right)}^{Ct_{\mathrm{ref}2}}}/{\left(1+E_{\mathrm{tar}}\right)}^{Ct_{\mathrm{tar}}}$. PCR efficiency (*E*) was calculated using standard curves corresponding to target and reference genes (*RPLP0* and *GAPDH*). Each standard curve included six points (diluted PCR product), with triplicate data obtained for each point. The expression of the reference genes did not change during the experiment. The primer sequences are shown in Table 1.

**Table 1:** Primers used in the study

Transcript	Strand	Sequence, 5′–3′	Product size, bp
*Atrogin‐1 (FBXO32)*	Forward Reverse	GTCCAAAGAGTCGGCAAGTC AGGCAGGTCAGTGAAGGTG	147
*TRIM63 (MURF1)*	Forward Reverse	CTCAGTGTCCATGTCTGGAGGCCGTT GGCCGACTGGAGCACTCCTGTTTGTA	147
*FOXO1*	Forward Reverse	TCCTACGCCGACCTCATC GCACGCTCTTGACCATCC	94
*DDIT4 (REDD1)*	Forward Reverse	GGTTTGACCGCTCCACGAG ATCCAGGTAAGCCGTGTCTTC	98
*RPLP0*	Forward Reverse	CACTGAGATCAGGGACATGTTG CTTCACATGGGGCAATGG	77
*GAPDH*	Forward Reverse	CAAGGTCATCCATGACAACTTTG GTCCACCACCCTGTTGCTGTAG	496

#### Western blot analysis

Frozen samples (~10 mg) were homogenized in ice‐cold RIPA buffer containing protease and phosphatase inhibitors (50 mmol/L *β*‐glycerophosphate, 50 mmol/L NaF, 1 mmol/L Na_3_VO_4_, 20 *μ*g/mL aprotinin, 50 *μ*g/mL leupeptin, 20 *μ*g/mL pepstatin, and 1 mmol/L PMSF). Samples were centrifuged for 10 min at 10,000*g* at 4°C. The supernatant was collected and stored at −80°C until analysis. Protein content was analyzed using the bicinchoninic acid assay. Samples were mixed with Laemmli buffer, loaded onto a 10% polyacrylamide gel (20 *μ*g protein per lane), and electrophoresis was performed using the Mini‐PROTEAN Tetra Cell system (Bio‐Rad) at 20 mA per gel. Proteins were transferred to nitrocellulose membranes for 30 min at 25 V using the Trans‐Blot Turbo system (Bio‐Rad). Membranes were stained with Ponceau S to verify protein transfer quality, followed by washing and incubation for 1 h in 5% nonfat dry milk. Next, membranes were incubated overnight at 4°C with anti‐TSC2^Thr1462^ (#3617; 1:2000), anti‐phospho‐p70S6K1^Thr389^ (#9205; 1:200), anti‐p70S6K1^Thr421/Ser424^ (#9204; 1:500), anti‐phospho‐4E‐BP1^Thr37/46^ (#2855; 1:1000), or anti‐phospho‐Erk1/2^Thr202/Tyr204^ (#4377; 1:500) (all from Cell Signaling Technology); and anti‐phospho‐FOXO1^Ser256^ (sc‐101681; 1:1000) (Santa Cruz Biotechnology); anti‐phospho‐ACC^Ser79^ (ab68191; 1:1000) or anti‐phospho‐eEF2^Thr56^ (ab115165; 1:1000) (both from Abcam).

The next day, membranes were incubated for 1 h with an HRP‐linked secondary antibody (Cell Signaling Technology) and washed with PBS‐Tween 20 after each step (three times for 5 min each). Following incubation of membranes with ECL substrate (Bio‐Rad), luminescent signals were captured using the ChemiDoc Imaging System (Bio‐Rad). Densitometry was performed using Image Lab 5.0 (Bio‐Rad). All values were expressed as the ratio of staining intensity for the target protein to the staining intensity of all proteins in corresponding electrophoresis lanes (in gels stained with Coomassie blue) as described previously (Ghosh et al. 2014).

#### Statistical analysis

Because the number of samples was small (*n* = 8), data are expressed as the median and IQR. For the same reason, no analysis of normality of distribution was carried out. Friedman's test with Dunn's multiple comparison was used to compare pre‐ and postexercise values. Differences between high‐intensity and moderate‐intensity exercise sessions were analyzed using the Mann–Whitney test. The level of significance was set at *P* ˂ 0.05. Statistical analysis was performed using GraphPad Prism 7 software (GraphPad Software).

## Results

In all volunteers, the 1RM for each leg was similar. The work volume performed by the leg moving a moderate load was 32% higher (*P* ˂ 0.001) than that of the contralateral leg moving a high load (Table 2). Blood lactate levels increased more than 10 times after cessation of exercise (*P* ˂ 0.001). Blood cortisol levels increased after 15 min of recovery (*P* ˂ 0.001), whereas blood testosterone levels did not change (Table 3).

**Table 2:** Strength exercise repetitions and work volume

Leg	Leg press 1RM, kg	Number of repetitions performed per set	Total work volume, (reps × sets × load)[kg]
L(65% 1RM)	204 [185–225]	15 [14–16]	9869 [8528–11,583]
H(85% 1RM)	207 [187–227]	9 [8–10]^a^	7461 [6375–8879]^a^

Each value represents the median and interquartile range.

a Denotes significant differences between the legs.

**Table 3:** Venous blood lactate, cortisol, and testosterone levels before, immediately after, and 15 min after termination of the exercise session

	Before	Immediately after	15 min after
Lactate, mmol/L	1.1 [0.8–1.6]	10.8 [9.8–12.4]^a^	8.3 [7.3–8.7]^a^
Cortisol, nmol/L	345 [201–532]	394 [258–599]	515 [358–694]^a^
Testosterone, ng/mL	10.9 [6.2–15.8]	10.3 [6.9–19.4]	7.9 [5.7–16.7]

Each value represents the median and interquartile range.

a Denotes significant differences from initial levels.

**Figure 1:**
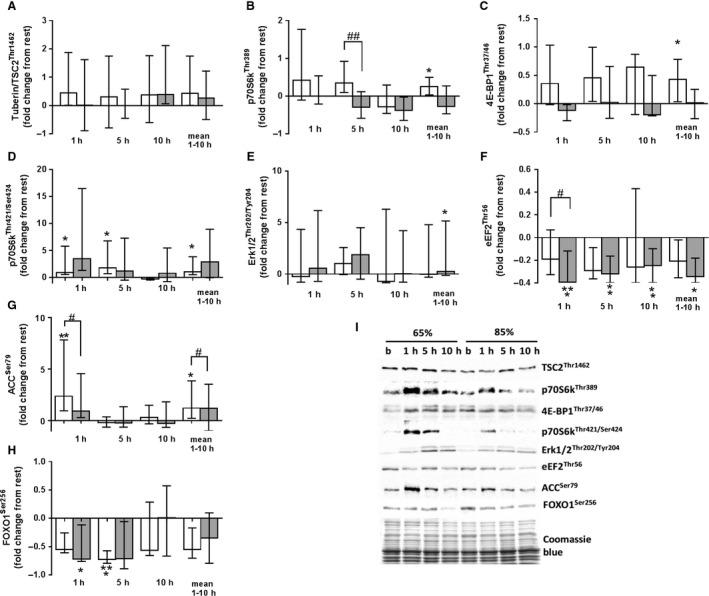
Fold changes (from rest) in expression of phosphorylated TSC2^Thr1462^ (a), p70S6k^Thr389^ (b), 4EBP1^Thr37/46^ (c), p70S6k^Thr421/Ser424^ (d), ERK1/2^Thr202/Tyr204^ (e), eEF2^Thr56^ (f), ACC^Ser79^ (g), and FOXO1^Ser259^ (h) in skeletal muscle at 1, 5, and 10 h after high load (shaded column; 85% 1RM) and moderate load exercise (white column; 65% 1RM). Representative immunoblots of the above are shown in (i). Data represent the median and interquartile range. * denotes a significant difference from pre‐exercise levels (*P < 0.05, **P < 0.01, ***P < 0.001). # denotes a significant effect of exercise load (^#^ *P *< 0.05, ^##^ *P *< 0.01).

**Figure 2:**
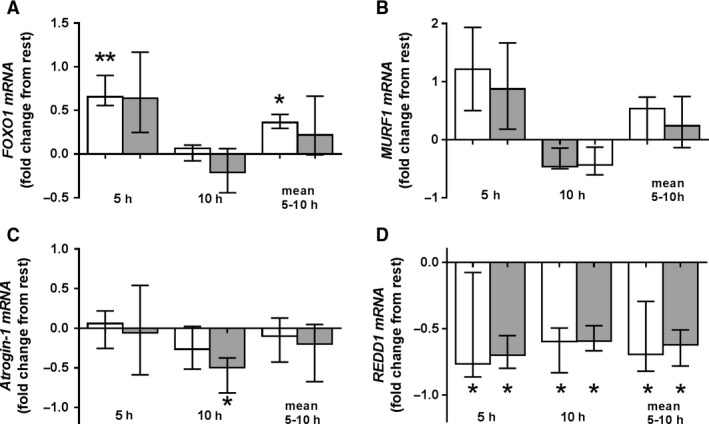
Fold changes (from rest) in expression of *FOXO1* (a), *TRIM63* [*MURF1*] (b), *FBXO32* [*Atrogin‐1*] (c), and *DDIT4* [*REDD1*] (d) mRNA in skeletal muscle at 5 and 10 h after high load (shaded column; 85% 1RM) and moderate load exercise (white column; 65% 1RM). Data represent the median and interquartile range. * denotes significant differences from pre‐exercise levels (*P *< 0.05). # denotes a significant effect of exercise load (*P *< 0.05).

Phosphorylation of tuberin (TSC2^Thr1462^) did not change after exercise under moderate or high load, with no differences between exercises (Fig. 1a). Phosphorylation of the p70 ribosomal protein S6 kinase (p70S6k^Thr389^), taken as an average during the recovery time after exercise, increased only after exercise under moderate load (*P* ˂ 0.05; Fig. 1b). However, there was a significant difference in phosphorylation of p70S6k^Thr389^ between the moderate and high load legs at 5 h postexercise (*P* ˂ 0.05). Phosphorylation of the 4E‐binding protein 1 (4E‐BP1^Thr37/46^) [as an average measured over the time of recovery] increased only after exercise under moderate load (*P* ˂ 0.05; Fig. 1c), whereas phosphorylation of p70S6k^Thr421/Ser424^ increased at 1 and 5 h after exercise under moderate load (*P* ˂ 0.05; Fig. 1d). By contrast, phosphorylation of extracellular signal‐regulated kinases 1 and 2 (ERK1/2^Thr202/Tyr204^) [as an average measured over the time of recovery] increased only after exercise under a high load (*P* ˂ 0.05; Fig. 1e). Phosphorylation of translation elongation factor 2 (eEF2^Thr56^) fell significantly at 1 (*P* ˂ 0.001), 5 (*P* ˂ 0.01), and 10 (*P* ˂ 0.01) h after cessation of exercise under high load (Fig. 1f). At 1 h after cessation of exercise, phosphorylation of eEF2^Thr56^ differed significantly according to load (*P* ˂ 0.05). Phosphorylation of acetyl‐CoA carboxylase (ACC^Ser79^) increased significantly at 1 h after termination of exercise under moderate load (*P* ˂ 0.01; Fig. 1g); this increase was significantly greater than that observed after exercise under high load (*P* ˂ 0.05). Phosphorylation of forkhead box O1 (FOXO1^Ser256^) fell significantly after exercises under both loads [5 h after moderate load exercise (*P* ˂ 0.001) and 1 h after high load exercise (*P* ˂ 0.05)] (Fig. 2h).

Expression of the *FOXO1* gene increased 5 h after exercise under moderate load (*P* ˂ 0.01; Fig. 2a). There were no significant differences in expression of *tripartite motif containing gene 63* (*TRIM63* also known as *MURF1*) under either load (Fig. 2b). Expression of the *F‐box protein 32* gene (*FBXO32,* also known as *Atrogin‐1*) fell at 10 h post‐exercise with a load of 85% 1RM (*P* ˂ 0.05; Fig. 2c). Expression of the *DNA damage inducible transcript 4* gene (*DDIT4*, also known as *REDD1*) fell at 5 and 10 h after exercises under moderate and high load (Fig. 2d).

## Discussion

The aim of this study was to examine activation of signaling molecules that regulate protein synthesis at the level of translation initiation and elongation, as well proteolysis in human skeletal muscle after exercise to volitional fatigue under moderate and high load. We found that phosphorylation of mTORC1 substrates increased after moderate load exercises, while phosphorylation of ERK1/2^Thr202/Tyr204^ increased, and that of eEF2^Thr56^ decreased, after high load exercises. These findings suggest that high and moderate load strength exercises performed to failure induce activation of different signaling cascades.

We chose to enroll strength athletes because signaling responses in trained muscles in response to strength exercises are more specific than those in untrained muscles (Raue et al. 2012). In addition, strength athletes possess well‐developed neural control and can recruit more muscle fibers during exercise under high load (Gabriel et al. 2006). It was shown that muscle hypertrophy is the result of accumulated increases in myofibrillar protein synthesis after resistance exercise mainly after attenuation of muscle damage (Damas et al. 2016). Thus, comparable muscle hypertrophy after exercise to fatigue with various intensities in untrained muscle can be associated not only with comparable activation of anabolic signaling, but also with the pronounced exercise‐induced injuries, as well as with efficiency of recovery. Additionally, Damas and co‐authors showed that exercise‐induced muscle damage after 3 weeks of training is much less pronounced than before training (Damas et al. 2016). We assume that in the trained skeletal muscle, in contrast with untrained, activation of anabolic signaling is associated with changes in the rate of protein synthesis after acute exercise and with regular training‐induced hypertrophy. That is why volunteers, who regularly use strength exercises for legs, including the leg press, were recruited in our study.

We found that levels of cortisol and lactate, both markers of exercise‐induced stress (Kraemer and Ratamess 2005; Fragala et al. 2011), increased; this may be related to the large amount of work performed and the marked levels of exercise‐induced stress. At the same time, volunteers performed both high load and moderate load strength exercises in a single exercise session, i.e., the potential influence of system factors on signaling processes in muscle was identical.

The work volume performed during moderate load exercises was higher than that during high load exercises. This may have led to more pronounced metabolic changes in muscle after moderate load exercise. This was confirmed indirectly by a more pronounced increase in phosphorylation of ACC^Ser79^, a well‐known marker of AMP‐activated protein kinase (AMPK) activation (Ha et al. 1994), after moderate load exercises than after high load exercises. Thus, exercise under high and moderate loads differed not only in term of mechanical stimulus, but also in terms of the metabolic shift induced by exercise, which may lead to activation of different signaling cascades.

Activation of mTORC1 was detected only after exercise under moderate load. Moreover, phosphorylation of p70S6K^Thr389^ at 5 h after cessation of exercise was higher after moderate load exercise than after high load exercise. A previous study showed that in contrast with exercise performed at 90% 1RM to fatigue, exercise with a load of 30% 1RM to fatigue led to an increase in phosphorylation of p70S6k^Thr389^ and 4E‐BP1^Thr37/46^ at 4 h after termination of the exercise session in recreationally active men (Burd et al. 2010). In that study and the present study, the exercise sessions at different loads involved different total work volume. Another study revealed no differences in the activation of mTORC1 after high‐volume and high‐intensity exercises in the muscles of trained volunteers (Gonzalez et al. 2015), but high‐volume and high‐intensity loads were not carried out to volitional fatigue. In our study, activation of mTORC1 depends on total work volume, rather than exercise load. These results are in agreement with studies in animal models (Ogasawara et al. 2017) and recreationally active men (Terzis et al. 2010) reporting dependence of changes in the magnitude of mTORC1 activation on total work volume.

The protein complex tuberin‐hamartine (TSC1/TSC2) is an important negative regulator of mTORC1 activity (Huang and Manning 2009). Therefore, we investigated pathways that activate (via *REDD1* gene expression (Wolff et al. 2011) and AMPK activation (Huang and Manning 2009)) and negatively regulate TSC1/TSC2 activity (via phosphorylation of AKT1‐dependent site TSC2^Thr1462^ (Manning et al. 2002)). We noted significant differences in phosphorylation of ACC^Ser79^ after high load and moderate load exercises. However, in contrast with that after moderate load exercises, AMPK activation after high load exercises was not observed. Expression of *REDD1* and phosphorylation of TSC2^Thr1462^ did not differ between high or moderate load exercises. Thus, lack of mTORC1 activation after high load exercise was not associated with activation of TSC1/TSC2.

Burd et al. (2010) did not observe activation of mTORC1 after a high load exercise; however, the mixed fractional synthesis rate increased to values comparable with those after low load exercises after which mTORC1 activation was observed in recreationally active humans. Thus, mTORC1 is not the only regulator that mediates increases in protein synthesis rate after strength training. A previous study shows that maximum electrostimulation‐induced contractions of rodent muscle activated mTORC1 as well as a number of mitogen‐activated protein kinases (MAPKs) and Ca^2+^/calmodulin‐dependent protein kinases (CAMKs) (Potts et al. 2017). To identify possible reasons for comparable activation of the protein synthesis rates after strength exercise under different loads, we analyzed activation of signal cascades potentially involved in regulating the rate of protein synthesis.

ERK1/2 activates mTORC1 by inhibiting the TSC1/TSC2 complex, and activates translation initiation and elongation by activating p90 ribosomal S6 kinase (p90RSK) (Proud 2007). eEF2 is the key regulator of translation elongation, and its activity increases as phosphorylation of Threonine 56 (Proud 2007) decreases. A decrease in the phosphorylation of eEF2^Thr56^ can be mediated by different mechanisms, including the ERK1/2‐p90RSK‐eEF2k and p70S6k‐eEF2k pathways (Wang et al. 2001). In our study, we detected an increase in ERK1/2^Thr202/Tyr204^ only after the high load exercises. In addition, a prolonged (up to 10 h recovery) fall in phosphorylation of eEF2^Thr56^ was observed after high load exercises, and differences after high load and moderate load exercises were significant. The parallel increase in phosphorylation of ERK1/2^Thr202/Tyr204^ and decrease in phosphorylation of eEF2^Thr56^ after the high load exercise may be evidence of ERK1/2‐p90RSK‐eEF2k‐dependent activation of eEF2. Thus, the absence of mTORC1 activation after high load exercise was compensated for by an increase in phosphorylation of ERK1/2^Thr202/Tyr204^ and a decrease in phosphorylation eEF2^Thr56^.

In contrast with our study, Burd et al. (2010) did not observe an increase in phosphorylation of ERK1/2^Thr202/Tyr204^ and a decrease in phosphorylation of eEF2^Thr56^ after a high load exercise in recreationally active men. The power lifters that participated in our study usually perform exercises at near maximal weight when training and competing. Training with high loads, unlike training with moderate loads, leads to a greater increase in strength due to an increase in specific force (Schoenfeld et al. 2017). A previous study shows that training with submaximal weight contributes to development of neuromuscular adaptation in trained people (Hakkinen et al. 1985). We assume that the reason for the difference in phosphorylation of ERK1/2^Thr202/Tyr204^ and eEF2^Thr56^ during high load exercises between the findings of our study and that of Burd et al. (2010) is training status of the participants. These changes in our study are probably due to higher muscle activation and greater mechanical stimulus in power lifters. In addition, there were differences between their study and ours in terms of the timing of biopsy sampling and diet regime. In another study, the same activation of MEK‐ERK1/2 signaling was shown both after exercises with moderate (60–65% 1RM) and high (80–85% 1RM) intensities in the muscles of untrained volunteers (Taylor et al. 2012). Importantly, in this paper, both moderate and high intensity exercises were not performed until volitional fatigue, besides that the volume of the total work performed and training status of volunteers significantly differed from our study.

Muscular hypertrophy, which is induced by strength training, depends both on the rate of protein synthesis and on the rate of proteolysis. Important regulators of ubiquitin‐dependent and lysosomal‐mediated protein degradation are FOXO transcription factors (Sandri et al. 2004; Zhao et al. 2007). In our study, we observed decreased phosphorylation of FOXO1^Ser256^ (an activation marker (Brunet et al. 1999)) after both types of exercise. This may be due to the fact that activation of FOXO1^Ser256^ is regulated by systemic factors such as insulin (van der Vos and Coffer 2011). Expression of the FOXO1‐dependent gene *MURF1* did not change after exercise under either load, whereas that of *FOXO1* increased significantly only after moderate load exercise. Since systemic factors in both cases had the same effect on signaling processes, it is possible that an increase in *FOXO1* expression was due to activation of AMPK after moderate load exercise. A previous study reported a link between AMPK activation and increased expression of *FOXO1* (Lee et al. 2015). In our study, expression of the E3‐ubiquitin ligase *Atrogin‐1*, which is also regulated by AMPK, fell after high load exercise. Since this occurred 10 h after the cessation of exercise, it is likely not related to changes of AMPK activation observed 1 h after cessation of exercise.

In previous studies, the effects of moderate and high intensity exercises on activation of the MEK‐ERK1/2 cascade (Taylor et al. 2012), on regulation of myogenesis (Wilborn et al. 2009) in the muscle of recreationally active men, and on activation of mTORC1 in muscle of strength trained men (Gonzalez et al. 2015) were evaluated. There were no differences in signaling activation and expression of genes regulating myogenesis after different exercises in these studies. In contrast with our work, in these studies, two exercise sessions were separated by a week. Therefore, the physiological state of the volunteers during the first and second exercise session could be different. Our experimental model allowed us to identify differences between the exercises with different intensities, as they were performed with the identical influence of systemic factors.

## Conclusions

In conclusion, exercise performed to volitional fatigue under a moderate load and a high work volume activated mTORC1‐dependent signaling, whereas exercise under a high load but a lower work volume resulted in activation of the MEK‐ERK1/2 signaling cascade and eEF2, in trained skeletal muscle. Expression of markers of ubiquitin‐proteasome system activation did not differ after high or moderate load exercise. Based on these data, we suggest that activation of mTORC1 after strength training is dependent on the work volume, whereas activation of MEK‐ERK1/2 and eEF2 is dependent on the load. Data showing comparable rates of protein synthesis observed after a single exercise session under different loads, and comparable hypertrophy levels after strength training under different loads performed to volitional fatigue, can be explained by activation of different signaling pathways that affect the level of translation initiation and elongation.

## Conflict of Interest

The authors declare that they have no conflict of interest.

## References

[phy214100-bib-0001] Brunet, A. , A. Bonni , M. J. Zigmond , M. Z. Lin , P. Juo , L. S. Hu , et al. 1999 Akt promotes cell survival by phosphorylating and inhibiting a forkhead transcription factor. Cell 96:857–868. 10.1016/S0092-8674(00)80595-4. 10102273

[phy214100-bib-0002] Burd, N. A. , D. W. D. West , A. W. Staples , P. J. Atherton , J. M. Baker , R. Daniel , et al. 2010 Low‐load high volume resistance exercise stimulates muscle protein synthesis more than high‐load low volume resistance exercise in young men. PLoS ONE 5:e12033 10.1371/journal.pone.0012033. 20711498 PMC2918506

[phy214100-bib-0003] Damas, F. , S. M. Phillips , C. A. Libardi , F. C. Vechin , M. E. Lixandrão , R. Jannig , et al. 2016 Muscle protein synthesis, hypertrophy, and muscle damage in humans. J. Physiol. 594:5209–5222. 10.1113/JP272472.This. 27219125 PMC5023708

[phy214100-bib-0004] Drummond, M. J. , C. S. Fry , E. L. Glynn , H. C. Dreyer , S. Dhanani , K. L. Timmerman , et al. 2009 Rapamycin administration in humans blocks the contraction‐induced increase in skeletal muscle protein synthesis. J. Physiol. 587(Pt 7):1535–1546. 10.1113/jphysiol.2008.163816. 19188252 PMC2678224

[phy214100-bib-0005] Fragala, M. S. , W. J. Kraemer , C. R. Denegar , C. M. Maresh , A. M. Mastro , and J. S. Volek . 2011 Neuroendocrine‐immune interactions and responses to exercise. Sports Med. 41:621–639. 10.2165/11590430-000000000-00000. 21780849

[phy214100-bib-0006] Gabriel, D. , G. Kamen , and G. Frost . 2006 Neural adaptations to resistive exercise: mechanisms and recommendations for training practices. Sports Med. 36:133–149. 16464122 10.2165/00007256-200636020-00004

[phy214100-bib-0007] Ghosh, R. , J. E. Gilda , A. V. Gomes , and M. Biology . 2014 The necessity of and strategies for improving confidence in the accuracy of western blots. Expert Rev. Proteomics 11:549–560. 10.1586/14789450.2014.939635.The. 25059473 PMC4791038

[phy214100-bib-0008] Gonzalez, A. M. , J. R. Hoffman , J. R. Townsend , A. R. Jajtner , C. H. Boone , K. S. Beyer , et al. 2015 Intramuscular anabolic signaling and endocrine response following high volume and high intensity resistance exercise protocols in trained men. Physiol. Rep. 3:1–15. 10.14814/phy2.12466. PMC4552541 26197935

[phy214100-bib-0009] Goodman, C. A . (2014). The role of mTORC1 in regulating protein synthesis and skeletal muscle mass in response to various mechanical stimuli Pp. 43–95 *in* Reviews of Physiology, Biochemistry and Pharmacology. Vol. 166 Springer, Cham. 10.1007/112_2013_17 24442322

[phy214100-bib-0010] Ha, J. , S. Daniel , S. S. Broyles , and K. Kim . 1994 Critical phosphorylation sites for acetyl‐CoA carboxylase activity. J. Biol. Chem. 269:22162–22168. 7915280

[phy214100-bib-0011] Hakkinen, K. , M. Alen , and P. V. Komi . 1985 Changes in isometric force‐ and relaxation‐time, electromyographic and muscle fibre characteristics of human skeletal muscle during strength training and detraining. Acta Physiol. Scand. 125:573–585. 4091001 10.1111/j.1748-1716.1985.tb07760.x

[phy214100-bib-0012] Hayot, M. , A. Michaud , C. Koechlin , P. Leblanc , F. Maltais , and C. Pre . 2005 Skeletal muscle microbiopsy: a validation study of a minimally invasive technique. Eur. Respir. J. 25:431–440. 10.1183/09031936.05.00053404. 15738285

[phy214100-bib-0013] Huang, J. , and B. D. Manning . 2009 The TSC1–TSC2 complex: a molecular switchboard controlling cell growth. Biochem. J. 290:1717–1721. 10.1042/BJ20080281.The. PMC2735030 18466115

[phy214100-bib-0014] Kraemer, W. J. , and N. A. Ratamess . 2005 Hormonal responses and adaptations to resistance exercise and training. Sports Med. 35:339–361. 15831061 10.2165/00007256-200535040-00004

[phy214100-bib-0015] Kumar, V. , A. Selby , D. Rankin , R. Patel , P. Atherton , W. Hildebrandt , et al. 2009 Age‐related differences in the dose‐response relationship of muscle protein synthesis to resistance exercise in young and old men. J. Physiol. 587:211–217. 10.1113/jphysiol.2008.164483. 19001042 PMC2670034

[phy214100-bib-0016] Lee, K. , E. Ochi , H. Song , and K. Nakazato . 2015 Activation of AMP‐activated protein kinase induce expression of FoxO1, FoxO3a, and myostatin after exercise‐induced muscle damage. Biochem. Biophys. Res. Comm. 466:289–294. 10.1016/j.bbrc.2015.08.126. 26342801

[phy214100-bib-0017] Manning, B. D. , A. R. Tee , M. N. Logsdon , J. Blenis , and L. C. Cantley . 2002 Identification of the tuberous sclerosis complex‐2 tumor suppressor gene product tuberin as a target of the phosphoinositide 3‐kinase/Akt pathway. Mol. Cell 10:151–162. 10.1016/S1097-2765(02)00568-3. 12150915

[phy214100-bib-0018] Mitchell, C. J. , T. A. Churchward‐Venne , D. W. D. West , N. A. Burd , L. Breen , S. K. Baker , et al. 2012 Resistance exercise load does not determine training‐mediated hypertrophic gains in young men. J. Appl. Physiol. 113:71–77. 10.1152/japplphysiol.00307.2012. 22518835 PMC3404827

[phy214100-bib-0019] Morton, R. W. , S. Y. Oikawa , C. G. Wavell , N. Mazara , C. Mcglory , J. Quadrilatero , et al. 2016 Neither load nor systemic hormones determine resistance training‐mediated hypertrophy or strength gains in resistance‐trained young men. J. Appl. Physiol. 121:129–138. 10.1152/japplphysiol.00154.2016. 27174923 PMC4967245

[phy214100-bib-0020] Ogasawara, R. , Y. Arihara , J. Takegaki , K. Nakazato , and N. Ishii . 2017 Relationship between exercise volume and muscle protein synthesis in a rat model of resistance exercise. J. Appl. Physiol. 123:710–716. 10.1152/japplphysiol.01009.2016. 28729395

[phy214100-bib-0021] Popov, D. , D. Tsvirkun , A. Netreba , O. Tarasova , A. Prostova , I. Larina , et al. 2006 Hormonal adaptation determines the increase in muscle mass and strength during low‐intensity strength training without relaxation. Human Physiol. 32:121–127. 17100349

[phy214100-bib-0022] Potts, G. K. , R. M. Mcnally , R. Blanco , J. You , A. S. Hebert , M. S. Westphall , et al. 2017 A map of the phosphoproteomic alterations that occur after a bout of maximal‐intensity contractions Key points. J. Physiol. 595:5209–5226. 10.1113/JP273904. 28542873 PMC5538225

[phy214100-bib-0023] Proud, C. G. 2007 Signalling to translation: how signal transduction pathways control the protein synthetic machinery. Biochem. J. 403:217–234. 10.1042/BJ20070024. 17376031

[phy214100-bib-0024] Raue, U. , T. A. Trappe , S. T. Estrem , H. Qian , L. M. Helvering , R. C. Smith , et al. 2012 Transcriptome signature of resistance exercise adaptations : mixed muscle and fiber type specific profiles in young and old adults. J. Appl. Physiol. 112:1625–1636. 10.1152/japplphysiol.00435.2011. 22302958 PMC3365403

[phy214100-bib-0025] Sandri, M. , C. Sandri , A. Gilbert , C. Skurk , E. Calabria , A. Picard , et al. 2004 Foxo transcription factors induce the atrophy‐related ubiquitin ligase atrogin‐1 and cause skeletal muscle atrophy. Cell 117:399–412. 10.1016/S0092-8674(04)00400-3. 15109499 PMC3619734

[phy214100-bib-0026] Schoenfeld, B. J. , M. D. Peterson , D. Ogborn , B. Contreras , and G. T. Sonmez . 2015 Effects of low‐vs. high‐load resistance training on muscle strength and hypertrophy in well‐trained men. J. Strength Cond. Res. 29:2954–2963. 10.1519/jsc.0000000000000958. 25853914

[phy214100-bib-0027] Schoenfeld, B. J. , J. Grgic , D. Ogborn , and J. W. Krieger . 2017 Strength and hypertrophy adaptations between low‐ versus high‐load resistance treining: a systematic review and meta‐analysis. J. Strength Cond. Res. 48:361–378. 10.1007/s40279-017-0795-y. 28834797

[phy214100-bib-0028] Taylor, L. , C. Wilborn , R. B. Kreider , and D. S. Willoughby . 2012 Effects of resistance exercise intensity on extracellular signal‐regulated kinase 1/2 mitogen‐activated protein kinase activation in men. J. Strength Cond. Res. 26:599–607. 22343976 10.1519/JSC.0b013e318242f92d

[phy214100-bib-0029] Terzis, G. , G. Georgiadis , G. Stratakos , I. Vogiatzis , S. Kavouras , P. Manta , et al. 2008 Resistance exercise‐induced increase in muscle mass correlates with p70S6 kinase phosphorylation in human subjects. Eur. J. Appl. Physiol. 102:145–152. 10.1007/s00421-007-0564-y. 17874120

[phy214100-bib-0030] Terzis, G. , K. Spengos , H. Mascher , G. Georgiadis , P. Manta , and E. Blomstrand . 2010 The degree of p70 S6k and S6 phosphorylation in human skeletal muscle in response to resistance exercise depends on the training volume. Eur. J. Appl. Physiol. 110:835–843. 10.1007/s00421-010-1527-2. 20617335

[phy214100-bib-0031] van der Vos, K. E. , and P. J. Coffer . 2011 The extending network of FOXO transcriptional target genes. Antioxid. Redox Signal. 14:579–592. 10.1089/ars.2010.3419. 20673124

[phy214100-bib-0032] Wang, X. , W. Li , M. Williams , N. Terada , D. R. Alessi , and C. G. Proud . 2001 Regulation of elongation factor 2 kinase by p90 RSK1 and p70 S6 kinase. EMBO J. 20:4370–4379. 10.1093/emboj/20.16.4370. 11500364 PMC125559

[phy214100-bib-0033] Wilborn, C. , L. Taylor , M. Greenwood , R. Kreider , and D. Willoughby . 2009 Effects of different intensities of resistance exercise on regulators of myogenesis. J. Strength Cond. Res. 23:2179–2187. 19826309 10.1519/JSC.0b013e3181bab493

[phy214100-bib-0034] Wolff, N. C. , S. Vega‐Rubin‐de‐Celis , X.‐J. Xie , D. H. Castrillon , W. Kabbani , and J. Brugarolas . 2011 Cell‐type‐dependent regulation of mTORC1 by REDD1 and the tumor suppressors TSC1/TSC2 and LKB1 in response to hypoxia. Mol. Cell. Biol. 31:1870–1884. 10.1128/MCB.01393-10. 21383064 PMC3133225

[phy214100-bib-0035] Zhao, J. , J. J. Brault , A. Schild , P. Cao , M. Sandri , S. Schiaffino , et al. 2007 FoxO3 coordinately activates protein degradation by the autophagic/lysosomal and proteasomal pathways in atrophying muscle cells. Cell Metab. 6:472–483. 10.1016/j.cmet.2007.11.004. 18054316

